# Missed opportunities: the detection and management of at-risk drinking and illicit drug use in acutely hospitalized patients

**DOI:** 10.3389/adar.2025.14149

**Published:** 2025-03-05

**Authors:** Danil Gamboa, Saranda Kabashi, Benedicte Jørgenrud, Anners Lerdal, Gudmund Nordby, Stig Tore Bogstrand

**Affiliations:** ^1^ Department of Medicine, Lovisenberg Diaconal Hospital, Oslo, Norway; ^2^ Department of Forensic Sciences, Oslo University Hospital, Oslo, Norway; ^3^ Department of Public Health Science, Institute of Health and Society, Faculty of Medicine, University of Oslo, Oslo, Norway; ^4^ Research Department, Lovisenberg Diaconal Hospital, Oslo, Norway

**Keywords:** emergency department, alcohol use, illicit drug use, substance use assessment, internal medicine

## Abstract

At-risk alcohol and illicit drug use are risk factors for disease and in-hospital complications. This study investigated whether clinicians document substance use in the electronic records of acutely hospitalized internal medicine patients. Alcohol and illicit drug positive patients were identified using prospectively gathered substance use data from a study sample comprising 2,872 patients included from November 2016 to December 2017 at an internal medicine hospital in Oslo, Norway. These data were unknown to hospital staff. Whether physicians recorded quantitative substance use assessments and interventions was examined in patients with study-verified alcohol use in excess of low-risk guidelines (Alcohol Use Disorder Identification Test-4 scores [AUDIT-4] of ≥5 for women and ≥7 for men) and/or illicit drug use (one or more illicit drug detected by liquid chromatography-mass spectrometry [LC-MS] analysis). Among 548 study-verified alcohol-positive patients, physicians documented quantity and frequency (QF) of use in 43.2% (n = 237) and interventions in 22.0% (n = 121). Alcohol interventions were associated with harmful drinking (AUDIT-4 ≥9 points; adjusted odds ratio [AOR] = 4.87; 95% CI: 2.54–9.31; p < 0.001) and QF assessments (AOR = 3.66; 95% CI: 1.13–11.84; p = 0.02). Among 157 illicit-positive patients, drug use was described quantitatively in 34.4% (n = 54) and interventions in 26.0% (n = 40). The rate of quantitative alcohol and illicit drug use assessment by hospital physicians is poor, with a correspondingly low intervention rate. Important opportunities for attenuating or intervening in at-risk alcohol and illicit drug use are missed.

## Introduction

The burden of alcohol and illicit drug use on public health and hospital resources [1–3] underscores the necessity for assessing both drinking habits and illicit drug use among hospitalized patients. Hospital-level prevalence rates for risky or problematic drinking range from 16% to 26% [4], and 15.6% screened positive for illicit drug use in a 2013 study of patients presenting to an inner-city emergency department (ED) [5]. Hospitalizations thus provide critical opportunities for detecting and treating patients with substance use disorder. However, studies examining substance use assessments and interventions among hospitalized patients have revealed suboptimal rates of both. For hospitalized patients with interview-verified alcohol use disorder (AUD), alcohol consumption was documented in only 40%–57% of medical records, and merely 53% were referred to treatment [6]. Among hospitalized patients using illicit drugs, substance use disorders were documented in the patient records of 26% of cannabis users, and 61% and 65% of cocaine and opioid users, respectively [7].

Recent population-level research has demonstrated a dose-response relationship between all-cause mortality and alcohol consumption, even below levels classified as high-risk, with a threshold of around 100 g/week [8]. Nevertheless, studies of detection rates for alcohol consumption in hospitalized patients have primarily focused on high-risk drinking or dependence [6, 9, 10]. Although the adverse effects of drinking may manifest at consumption volumes lower than typically considered harmful, data regarding drinking habits at lower-risk levels in this population are notably sparse. Moreover, internal medicine patients are disproportionately vulnerable to the adverse effects of alcohol use compared to the general population due to factors including older age [11] and associated physiological decline [12], a higher degree of polypharmacy [13] and increased risk of alcohol-drug interactions [14, 15]. These facts highlight the need for examining how hospital physicians document and address a broader range of drinking habits.

Our aim was to determine the rate at which hospital physicians documented complete assessments of alcohol and illicit drug use. Using data from a study examining substance use in acutely hospitalized patients, we were able to investigate the extent of substance use documentation as recorded by hospital physicians in this population with study-verified alcohol and illicit drug use. In alcohol-positive patients, we also examined whether alcohol consumption was registered using diagnosis codes, and whether complete assessments and interventions were associated with various patient and hospital stay characteristics.

## Materials and methods

### Study design and sample

In this cross-sectional study, we analyzed previously collected data from a prospective study conducted in 2017 at Lovisenberg Diaconal Hospital in Oslo, Norway [16, 17] examining various dimensions of substance use in acutely hospitalized patients presenting to the emergency department. Alcohol consumption was measured by the Alcohol Use Disorder Identification Test-4 [18] and illicit drug use was detected through liquid chromatography – mass spectrometry (LC-MS) analysis [19] of whole blood. AUDIT-4 scores and LC-MS results served as our inclusion criteria when identifying patient records eligible for inclusion in our analysis, resulting in 688 instances of either alcohol- and/or illicit drug-positivity, from an original sample of 2,872 acutely-admitted internal medicine patients. Alcohol positivity was defined as an AUDIT-4 score of ≥5 points for women and ≥7 points for men, indicative of alcohol use in excess of low-risk guidelines [18]. Illicit drug positivity was defined as LC-MS detection of tetrahydrocannabinol (THC), amphetamine and methamphetamine, cocaine (through its metabolite benzoylecognin), methylenedioxy-methylamphetamine (MDMA/“ecstasy”), or heroin (determined through morphine/codeine-ratio) [20]. Importantly, study-obtained alcohol and illicit drug use rates were never at any point available to the hospital physicians. These rates thus served as the comparative standard when investigating our outcomes in the electronic patient records, namely substance use assessments.

All participants in the original study were 18 years or older and included via informed consent at all hours of the day throughout a 12-month period, with a participation rate of 81%. Study inclusion generally occurred upon admission to the ED, or shortly after transfer to a ward. Further methodological details have been described elsewhere [16, 17].

### Site characteristics and co-variates

In Norway, evaluation at an emergency department is defined as specialist healthcare, and patients must first undergo pre-hospital sorting before referral to an ED [21], thereby prioritizing hospital resources for the conditions that require them. Pre-hospital sorting is performed by family physicians, in emergency rooms staffed by general practitioners, or by emergency services. As ED admissions are indicative of more serious or acute illness, they are defined as hospitalizations in our sample. Patient trajectory was further divided into 1) ward admission following initial management in the ED, 2) evaluation and management in the ED as an outpatient, or 3) other (emergent transfer to another hospital, discharge against medical advice, or death).

Initial evaluation and management of patients presenting to the ED is documented in an admission record. The majority of patients receive further treatment at an appropriate ward, with accompanying discharge summaries. Patient documentation is limited to a single outpatient record if evaluation and management is concluded within 5 h of admission to the ED. Primary and secondary diagnosis codes using the International Classification of Diseases, 10th Edition (ICD-10) are registered upon discharge [22] by the physician.

Demographic data comprised age, gender, and occupational status. In addition to alcohol use, patients self-reported psychiatric symptoms using the Symptom Checklist-5 (SCL-5), a short-form screening tool where a score greater than 2 points indicates psychological distress [23]. Apart from illicit drug use, we also included any concurrent use of psychoactive medication in our analysis, defined as LC-MS detection of either benzodiazepines, z-hypnotics, or opioids.

Among alcohol-positive patients, we further identified AUDIT-4 scores of ≥9 points [18], indicative of harmful alcohol use and/or possible dependence. AUDIT-4 [18] is a validated self-reported screening tool for stratifying alcohol consumption, consisting of four items: drinking frequency, average number of standard drinks consumed per drinking episode, frequency of high-intake instances of drinking (≥4 drinks for women or ≥5 drinks for men per drinking episode), and any instance of concern regarding drinking habits from friends, family or medical professional. Scores range from 0 to 16 points.

### Alcohol and illicit drug use documentation

We reviewed patient records upon admission and discharge in order to compare physician-documented substance use assessments with study-obtained alcohol consumption patterns and illicit drug use. We defined a complete alcohol assessment as describing both quantity and frequency (QF) of alcohol use, such as units of alcohol consumed per week, or any measurement unit convertible to standard drinks (e.g., number of fixed-volume alcoholic containers) in a given time period.

Defining illicit drug positivity as detection in patient blood via LC-MS analysis precluded estimations of usage frequency for comparative purposes when examining patient records. Nevertheless, we considered illicit drug use frequency to be a clinically relevant metric, as it serves as an indicator of total drug usage burden. A complete illicit drug assessment was therefore defined as describing both the type of substance and frequency of use. Assessments were classified as incomplete if they employed only one of two objective measures or only qualitative descriptors (e.g., “drinks moderately;” “recreational drug use”).

In alcohol-positive patients, we measured the frequency of alcohol consumption-related ICD-10 diagnosis codes, which were F10.0-F10.7 (alcohol-related disorders, including harmful use), Z72.1 (alcohol use), Y91.0-Y91.3 and Y91.9 (degrees of alcohol intoxication), and T51.0 (toxic effects of ethanol).

Interventions were broadly defined as any recorded instance of 1) patient-directed counseling, 2) specific post-discharge follow-up with a family physician or 3) referral or transfer to a treatment institution for alcohol or substance use disorders.

### Statistical analysis

The study outcomes were the rates of complete physician-recorded QF assessments of alcohol consumption and complete illicit drug use assessments. Secondary outcomes in alcohol-positive patients were the rate of intervention and percentage with an alcohol-related ICD-10 diagnosis code upon discharge. Unadjusted outcome rates are presented in descriptive tables. Group differences across categorical variables were analyzed using *X* ^2^ statistics with associated p-values. Continuous variables are shown as means with standard deviations (SDs) if normally distributed and as medians with interquartile ranges if not-normally distributed. Using logistic regression, we estimated the relationship between alcohol-related study outcomes and co-variates. Our dependent binary variables were complete QF assessments of alcohol consumption versus incomplete or absent assessments, and any instance of intervention versus no recorded intervention. Significant associations in the univariate analyses were subsequently included in an adjusted model for each outcome variable. Based on the comparatively lower number of illicit drug positive instances eligible for inclusion, logistic regression analysis related to illicit drug use documentation was expected to be insufficiently powered. Estimates are presented as odds ratios with associated 95% confidence intervals, where a p-value < 0.05 is considered indicative of statistical significance. All statistical analyses were performed using IBM SPSS 25.0 (Armonk, NY). Cases with missing data were excluded.

### Patient and public involvement

There was no patient or public involvement in the design or execution of this study.

## Results

### Sample characteristics and study outcome rates

**FIGURE 1:**
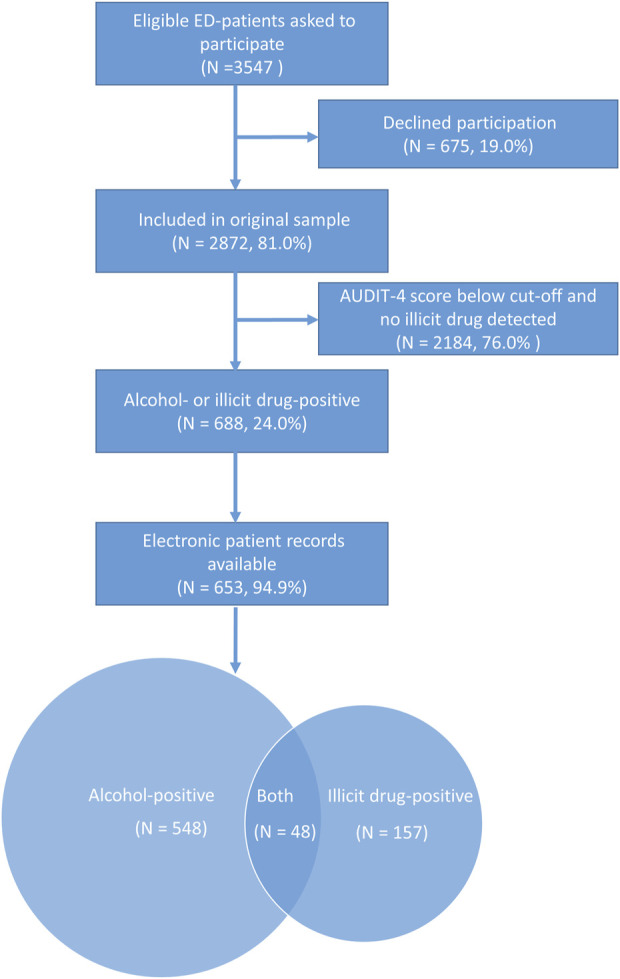
Sample inclusion. Legend: Flowchart detailing sample selection from the original sample of 2,872 patients admitted to the ED and included in the study. Alcohol-positive criteria for inclusion: AUDIT-4 score ≥5 for women and ≥7 for men. Illicit drug-positive criteria for inclusion: laboratory detection of at least one illicit substance. Among 688 patients eligible for inclusion, electronic patient records were available in 653 patients, with 548 alcohol-positive instances and 157 illicit drug-positive instances, with 48 patients positive for both.

Among the 688 patients eligible for inclusion in our analysis, electronic patient records were available in 653, comprising 548 instances of alcohol-positivity and 157 instances of illicit drug-positivity (Figure 1), with 48 patients being positive for both.

Sample characteristics and unadjusted study outcome rates are presented in Table 1. Among the patients testing positive for alcohol (i.e., AUDIT-4 scores indicating drinking above low-risk thresholds), physicians documented complete QF descriptions of alcohol consumption for less than half (43.2%), an intervention in less than a quarter (22.0%) and an alcohol-related ICD-10 diagnosis in less than 1 in 10 (9.1%). Among patients with illicit drug use identified through LC-MS detection in whole blood, physicians documented a complete illicit drug assessment in 34.4% of the patients’ medical records and an intervention was recorded in 26.0%.

**TABLE 1:** Characteristics and study outcome rates of patients positive for alcohol (AUDIT-4 score above low-risk) and/or illicit drugs (detected in blood), N = 653.

Characteristics	Alcohol-positive (n = 548)	Illicit drug-positive (n = 157)
Age^a^		
Median – yr (IQR^‡^)	46.0 (34.0)	45.0 (27)
Age ≥65 yr – n (%)	133 (24.3)	12 (7.6)
Female – n (%)	267 (48.7)	52 (33.3)
Occupational status^a^		
Working – n (%)	314 (57.3)	56 (35.7)
Retired – n (%)	221 (23.0)	14 (8.9)
Not working – n (%)	92 (16.8)	75 (47.8)
Psychological distress (SCL-5 score)^a^		
>2 points – n (%)	170 (31.0)	68 (43.3)
Type of stay^a^		
Ward admission – n (%)	416 (75.9)	103 (65.6)
Outpatient evaluation – n (%)	106 (19.3)	34 (21.7)
Other – n (%)	25 (4.6)	20 (12.7)
AUDIT-4 score		
Median – points (IQR^c^)	7.0 (4.0)	4.0 (6.0)
Harmful alcohol use (9 to 16 points) – n (%)	203 (37.0)	22 (14.0)
Alcohol consumption patterns		
Median grams per week – gram (IQR^c^)	105 (160.5)	
At least one binge per month – n (%)	461 (84.1)	
Illicit drug detected		
THC – n (%)	27 (4.9)	90 (57.3)
Other – n (%)	21 (3.8)	67 (42.7)
Psychoactive medication detected – n (%)	165 (30.0)	101 (64.3)
Study outcomes		
Alcohol use, complete assessment^b^ – n (%)	237 (43.2)	
Illicit drug use, complete assessment^b^ – n (%)		54 (34.4)
Alcohol-related ICD-10 diagnosis code – n (%)	50 (9.1)	
Intervention performed – n (%)	121 (22.0)	40 (26.0)

^a^ Number of cases with missing data for each variable is described in Supplementary Table S1.

^b^ Complete alcohol assessments defined as describing both quantity and frequency of alcohol consumption, and complete illicit drug assessment defined as describing type of substance and frequency of use.

^c^ Interquartile range.

### Assessment rates upon admission and discharge

**FIGURE 2:**
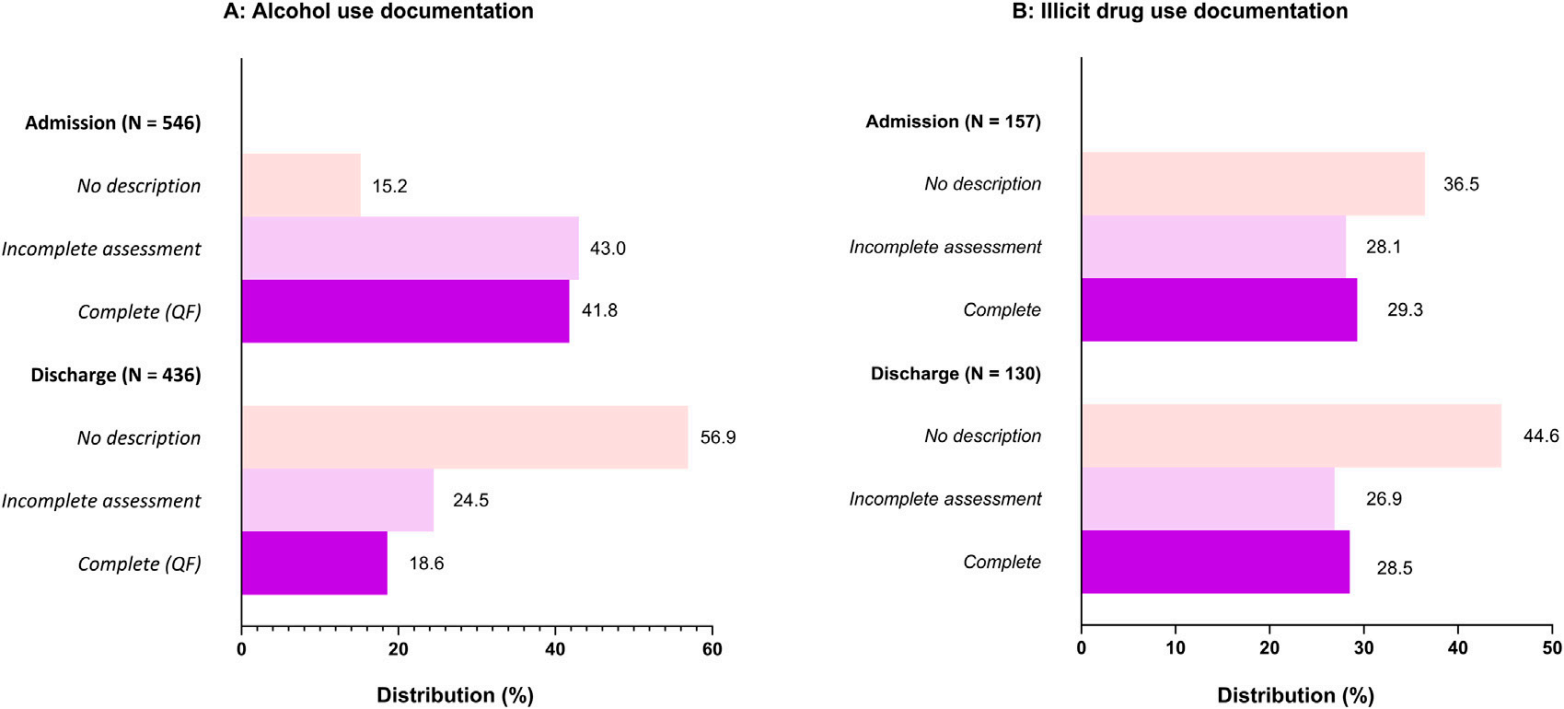
**(A)** Alcohol consumption assessments in the admission and discharge records of alcohol-positive patients. **(B)**: Illicit drug use assessment in the admission and discharge records of illicit drug-positive patients. Legend: Frequencies of different levels of alcohol and illicit drug use assessment at admission and discharge. Complete alcohol use assessments comprise quantity and frequency, and complete illicit drug use assessments comprise type of substance and frequency. Assessments are classified as incomplete if employing qualitative terms or only one of two objective descriptors. Admissions records encompass all patients presenting to the emergency department, including outpatient evaluations.

The rate at which physicians recorded alcohol assessments differed between admission and discharge (Figure 2A). Physicians documented complete QF assessments in 41.8% of admission records, whereas 43.0% contained incomplete assessments, and 15.2% lacked any alcohol use assessment. In the discharge records of alcohol-positive patients, only 18.6% included a complete QF assessment, 24.5% contained incomplete assessments, and alcohol consumption was not described at all in 56.9% of discharge records.

Corresponding rates among illicit drug-positive patients (Figure 2B) upon admission were complete drug use assessments in 31.2%, incomplete assessments in 29.9% and no assessment in 38.9%. In the discharge records of illicit drug-patients, complete assessments were present in 28.5%, incomplete assessments in 26.9%, and no description of illicit substance use in 44.6%. Variations in the distribution of illicit assessment degree across patient and hospital stay characteristics is presented in Supplementary Figure S1.

**FIGURE 3:**
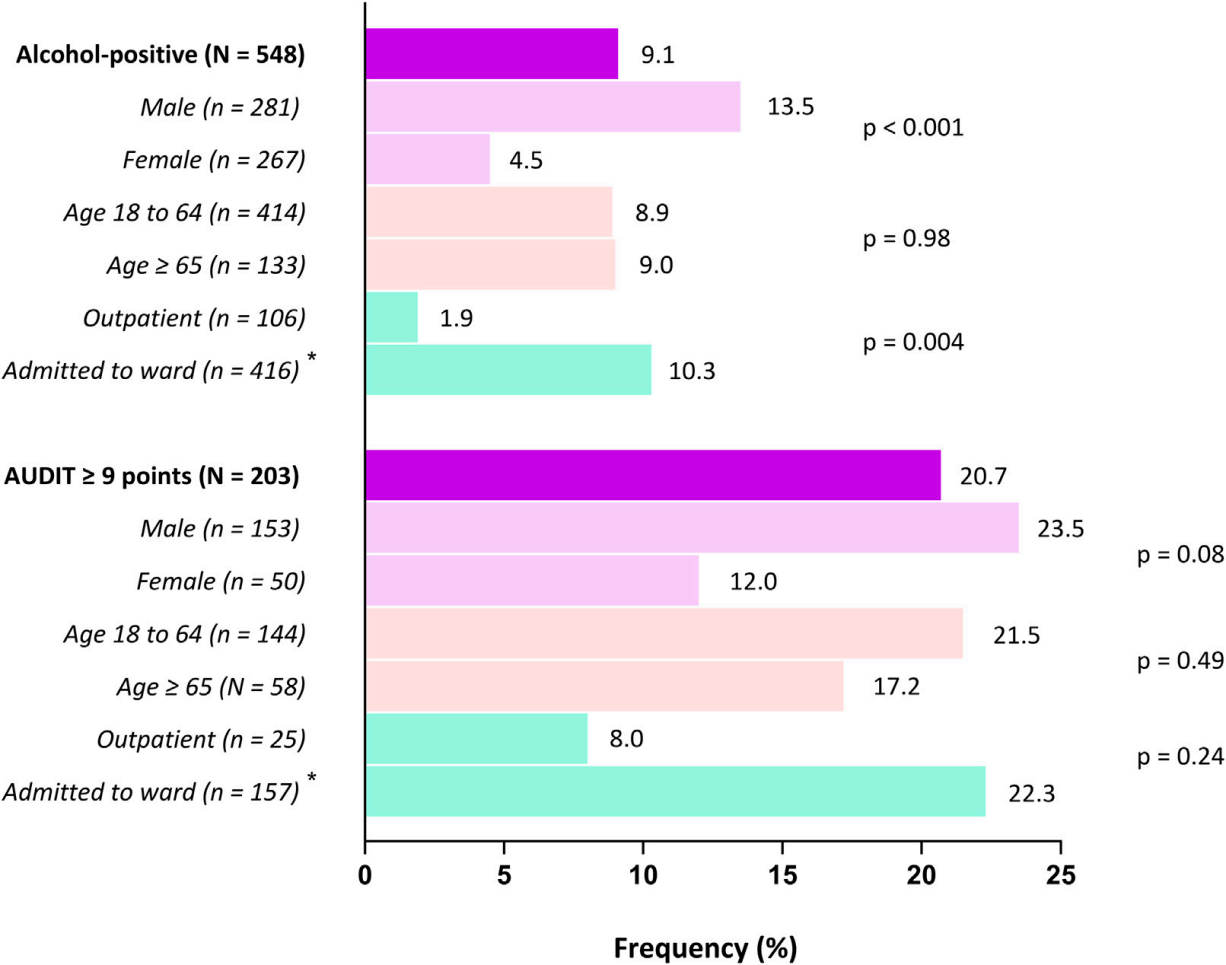
Frequency of alcohol-related ICD-10 diagnosis codes among alcohol-positive patients Legend: An alcohol-related ICD-10 diagnosis was defined as any instance of F10.0-F10.7 (alcohol-related disorders, including harmful use), Z72.1 (alcohol use), Y91.0-Y91.3 and Y91.9 (degrees of alcohol intoxication), or T51.0 (toxic effects of ethanol) as a primary or secondary diagnosis. Alcohol-positive patients comprised all those eligible for inclusion in the analysis based on their AUDIT-4 score (≥5 for women and ≥7 for men). *Rates for hospital stays classified as “other” not shown, but were included in the analysis.

Furthermore, among patients with harmful alcohol consumption (AUDIT-4 scores of ≥9 points), an alcohol-related ICD-10 diagnosis (Figure 3) was coded by physicians in 20.7%. Differences across gender (male, 23.5%; female, 12.0%; p = 0.08) and type of stay (admitted to ward, 22.3%; outpatient, 8.0%; p = 0.24) were non-significant.

### Patient characteristics associated with complete (quantity and frequency) assessments of alcohol consumption

In the univariate analysis (Table 2), the likelihood of patient records containing complete QF descriptions of alcohol consumption increased if harmful alcohol consumption (AUDIT-4 score of ≥9 points) was present (OR 1.97), for every 1 year increase in age (OR 1.04), and if patients were retired (OR 2.41) or not working (OR 2.05), and the likelihood decreased for outpatient evaluations (OR 0.33). In the multivariate analysis, associations with harmful alcohol consumption (OR 1.73), age (OR 1.05) and outpatient evaluations (OR 0.37) persisted. However, QF assessments were now less likely in retired patients (OR 0.48).

**TABLE 2:** Factors associated with documented assessment of both the quantity and frequency of alcohol consumption among alcohol-positive patients.

Characteristics	Unadjusted		Adjusted	
	Odds ratio (95% CI)	P-value	Odds ratio (95% CI)	P-value
*Age (year)*	1.04 (1.03–1.05)	<0.001	1.05 (1.03–1.07)	<0.001
*Female*	0.75 (0.54–1.06)	0.10	-	-
*Harmful alcohol use (AUDIT-4 ≥ 9 points)* ^a^	1.97 (1.38–2.80)	<0.001	1.73 (1.13–2.64)	0.01
*Psychological distress (SCL-5 > 2 points)*	1.07 (0.74–1.55)	0.72	-	-
*Occupational status*				
Working	Ref.		Ref.	
Retired	2.41 (1.57–3.70)	<0.001	0.48 (0.23–0.99)	0.05^*^
Not working	2.05 (1.28–3.28)	0.003	1.06 (0.59–1.93)	0.84
*Type of stay*				
Admitted to ward	Ref.		Ref.	
Outpatient	0.33 (0.20–0.54)	<0.001	0.37 (0.22–0.63)	<0.001
Other^b^	0.71 (0.31–1.62)	0.42	0.53 (0.20–1.42)	0.21
*Illicit drug detected*	0.63 (0.34–1.19)	0.16	-	-
*Psychoactive medication detected* ^c^	1.26 (0.88–1.82)	0.21	-	-

Unadjusted and adjusted estimates of association between complete QF assessments of alcohol consumption and patient- and hospital stay characteristics. Univariate analysis estimates with P-values < 0.05 were included in the multivariable model.

^a^ Ref: AUDIT-4 score ≥5–8 for women and ≥7-8 for men.

^b^ Emergent transfer to other hospital, discharge against medical advice, or death.

^c^ Laboratory detection of one more of either a benzodiazepine, opioid or z-hypnotic.

*P = 0.048.

### Factors associated with documented intervention among alcohol positive patients

In univariate analyses, physician-documented intervention (Table 3) was more likely among alcohol positive-patients with harmful (AUDIT-4 score ≥9 points) alcohol consumption (OR 7.36), a complete QF assessment of alcohol use (OR 4.26), a positive screen (SCL-5 >2 points) for psychological distress (OR 4.19), who were not working (OR 5.92), or who tested positive for one or more illicit drugs (OR 2.11) or psychoactive medicines (OR 2.06). In the multivariable model, documentation of intervention was more likely for alcohol-positive patients with complete QF assessments of alcohol use (OR 3.66), harmful alcohol consumption (OR 4.87), psychological distress (OR 2.57) and who were not working (OR 2.46), but was no longer associated with type of stay or the presence of one or more illicit drugs or psychoactive medicines.

**TABLE 3:** Factors assoiated with documented alcohol intervention.

Characteristics	Unadjusted		Adjusted	
	Odds ratio (95% CI)	P-value	Odds ratio (95% CI)	P-value
*Age (year)*	1.01 (1.00–1.02)	0.09	-	-
*Female*	0.39 (0.25–0.63)	<0.001	0.59 (0.31–1.10)	0.21
*Harmful alcohol use (AUDIT ≥ 9 points)* ^a^	7.36 (4.50–12.03)	<0.001	4.87 (2.54–9.31)	<0.001
*Alcohol assessment*				
None	Ref.		Ref.	
Incomplete^b^	1.76 (0.75–4.17)	0.20	1.31 (0.39–4.36)	0.66
Quantity and frequency	4.26 (1.86–9.74)	<0.001	3.66 (1.13–11.84)	0.03
*Psychological distress (SCL-5 >2 points)*	4.19 (2.64–6.66)	<0.001	2.57 (1.41–4.68)	<0.01
*Occupational status*				
Working	Ref.		Ref.	
Retired	1.45 (0.79–2.65)	0.23	0.76 (0.36–1.63)	0.49
Not working	5.92 (3.41–10.25)	<0.001	2.46 (1.21–4.97)	0.01
*Type of stay*				
Admitted to ward	Ref.		Ref.	
Outpatient	0.41 (0.21–0.82)	0.01	0.60 (0.23–1.55)	0.29
Other^c^	1.22 (0.47–3.16)	0.69	0.49 (0.15–1.57)	0.23
*Illicit drug detected*	2.11 (1.09–4.08)	0.03	1.17 (0.46–2.94)	0.74
*Psychoactive medication detected* ^d^	2.06 (1.32–3.22)	0.001	1.26 (0.68–2.33)	0.46

Legend: Unadjusted and adjusted estimates of association between any physician-documented intervention and level of alcohol use assessment and patient- and hospital stay characteristics. Univariate analysis estimates with P-values < 0.05 were included in the multivariable model.

^a^ Ref: AUDIT-4 score 5–8 for women and 7-8 for men.

^b^ Classified as incomplete if qualitative terms or only one of two quantitative descriptors are employed.

^c^ Emergent transfer to other hospital, discharge against medical advice, or death.

^d^ Laboratory detection of one more of either a benzodiazepine, opioid or z-hypnotic.

## Discussion

When comparing study data to patients’ electronic medical records, physicians documented alcohol use employing quantity and frequency in approximately 4 out of 10 patients with drinking habits exceeding low-risk guidelines, and in instances of harmful drinking, rarely coded this with an ICD-10 diagnosis. In the majority of discharge records, assessments of alcohol use were either absent or incomplete. The overall rate of alcohol-related intervention was low and was associated with a harmful level of drinking (based on self-report) and a physician’s documentation of a complete QF assessment of alcohol use. Among patients testing positive for an illicit drug, one in three had patient records documenting both the type of substance and frequency of use.

Our findings are congruent with earlier studies reporting suboptimal rates of both assessment and intervention of alcohol and substance use [7, 9]. While detection rates have previously generally been examined among patients with harmful levels of drinking or substance use disorders, this study has examined how physicians document alcohol and illicit drug use across a wider spectrum of usage patterns. Since AUDIT-4 scores and laboratory detection rates of illicit drugs were obtained from a concurrent study and thus unknown to hospital physicians, this allowed for a direct comparison with alcohol and drug use documentation in patient records.

Apart from diseases directly attributable to alcohol, such as alcoholic liver disease [24], drinking habits at risk levels not classified as overtly harmful still exert negative effects on several commonly encountered conditions in hospitalized patients. Clinicians should be aware of increased recurrence rates of atrial fibrillation [25], higher risk of readmission for heart failure [26], poorer glycemic control in patients with diabetes mellitus [27], and associations with falls in the older adults [28]. Furthermore, alcohol and substance use is associated with medication non-adherence [29], complicates anticoagulant therapy [30], and interacts negatively with psychoactive prescription medication [31]. Importantly, accurate and comprehensive substance use assessments are necessary in order to identify patients at risk for developing withdrawal syndromes [32], where delirium may be fatal [33].

Emergency departments are high-stress environments [34, 35], where time constraints likely impede certain elements of history taking, suggested by lower rates of alcohol use documentation and intervention among the outpatients in our sample. Nevertheless, a validated tool for stratifying alcohol consumption into different levels of risk is readily available through AUDIT-4 [18], which may be simplified further to comprise only quantity and frequency [36]. Notably, patients in our sample readily self-reported both at-risk and harmful levels of drinking during their inclusion in the original study, and hospital admissions have been highlighted as valuable opportunities for assessing and treating harmful levels of alcohol consumption. High-risk drinking patterns (≥5 units for men and ≥4 units for women at least once a week) [37] associated with the development of alcohol use disorder can be identified, and quantity and frequency measurements may be utilized in risk-communication when counseling patients on drinking habits, highlighting the dose-response relationship with reduction in life-expectancy [8]. As brief interventions in the ED appear to reduce at-risk drinking in the short term [38], an increase in screening rates can attenuate progression into overtly harmful alcohol use among a larger number of at-risk drinkers. The viability of targeting this group is supported by the proportionality between total volume of alcohol consumed and the rate of harmful alcohol use in population-level data [39].

Considerations regarding the paucity of complete illicit drug use documentation is limited by the lack of estimates of associated factors, as these analyses were under-powered. In the unadjusted analysis, complete illicit drug use assessments appeared to be more frequent in instances of psychological distress, whereas variations within other co-variates did not appear to persist from admission to discharge. Interpretability is further complicated by illegality as the defining substance group characteristic, which does not account for the heterogeneity in potential health risk associated with the various drugs. As the study-obtained illicit drug use rates consisted solely of whether a drug was present or not, comparable data regarding dosage and frequency akin to alcohol usage patterns were not available. Even so, it is conceivable that low rates of documentation, including the lack of usage frequencies in patient records, may have resulted in instances of harmful or otherwise clinically relevant drug use remaining undetected. The percentage of complete assessments was equally low in patients both positive for THC and any of the other drugs, possibly indicating patient or physician under-estimation, or harmful usage patterns being misattributed as “recreational.”

Patient records are foundational medical documents, and accurate, comprehensive and relevant discharge summaries provide essential information regarding future care [40], ensuring treatment continuity. Post-discharge trajectories are often managed by general practitioners, where absent or inadequate alcohol use assessments may lead to missed opportunities for managing at-risk or harmful drinking habits. For instance, alcohol interventions in primary healthcare settings result in a 12-month reduction in harmful drinking rates [41]. Alcohol and substance use assessments should therefore be documented in discharge summaries, akin to other clinically relevant information, such as tobacco use [42]. The utility of incomplete (i.e., qualitative or lacking either quantity or frequency) assessments of alcohol consumption is likely limited, as these were not associated with interventions, as opposed to QF assessments. Qualitative descriptors are also vulnerable to interpretative ambiguity when patient records are examined by future healthcare providers.

Alcohol-related harm in hospital populations has typically been identified through ICD-10 diagnoses, as determining alcohol consumption patterns by assessing patient records is resource-intensive. However, the low rate at which physicians coded an alcohol-related ICD-10 diagnoses among patients self-reporting harmful levels of drinking in our sample indicates that the extent of alcohol-related harm in hospital populations may be under-estimated. The representativeness of diagnosis code registries may improve by more consistent coding; ICD-10 diagnoses are not limited to only dependency syndromes, and also comprise harmful use, acute intoxications, and varying degrees of influence. Even so, clinician reticence due to perceived or concrete sociocultural barriers [43] may contribute to limited alcohol-related diagnostic coding. When counseling patients in order to prevent progression into harmful drinking or negative interactions with medication, clinicians may also employ the ICD-10 code simply denoting alcohol use without reference to harm.

Our study sample was culled from a large population of patients evaluated at a mid-sized urban hospital, where the original criteria for inclusion were wide. While our findings are in line with prior research demonstrating unsatisfactory substance use assessments, the single site origin of the data may reflect local documentation practices. The reliability of self-reported alcohol consumption may be affected by recall bias [44] and other factors, albeit usually in the direction of under-reporting [45]. Substance use assessments and less formal instances of alcohol or drug use counseling may have been performed without being recorded in the patient’s medical record.

While changing clinical practice is a demanding endeavor [46], efforts to improve the rate of substance use documentation in the acutely hospitalized should be encouraged. Integrating screening into electronic triage tools has shown promise, with screening rates approaching 97% [47], whereas physician-directed training has yielded more modest results [48]. The effect of improved alcohol and drug use assessments can be evaluated through a multi-center study, where, for example, drinking habits are measured before and after the implementation of standardized substance use assessments.

## Data Availability

The datasets presented in this article are not readily available because of an institutional agreement. Anonymized data is available from the corresponding author upon reasonable request. Requests to access the datasets should be directed to danil.gamboa@studmed.uio.no.

## Supplementary material

SUPPLEMENTARY FIGURE S1 Distribution of illicit drug use assessments across co-variates. Legend: Percentage distribution of the different levels of illicit drug use assessments at admission and discharge across patient and hospital stay characteristics. Within-group differences across co-variates were examined using X^2^-statistics, with associated p-values presented above the bar line.

## Abbreviations

AUD, alcohol use disorder; AUDIT-4, Alcohol Use Disorder Identification Test 4; CI, confidence interval; ED, emergency department; LC-MS, liquid chromatography-mass spectrometry; OR, odds ratio; QF, quantity and frequency; SCL-5, Symptom Checklist 5; SUD, substance use disorder.
