# Supplementary material for: Missed opportunities: the detection and management of at-risk drinking and illicit drug use in acutely hospitalized patients
Source: Adv Drug Alcohol Res. 2025 Mar 5;5:14149. doi: 10.3389/adar.2025.14149 (PMC11919628; doi:10.3389/adar.2025.14149)
Supplement: Supplementary file 1 [file Table1.DOCX]

| **Supplemental Table 1. Missing data in patient characteristics.** | | | |
| --- | --- | --- | --- |
|  | | **Alcohol-positive**  **(N = 548)** | **Illicit drug-positive**  **(N = 157)** |
| **Characteristics** | |  |  |
| Age | |  |  |
|  | Missing – no. (%) | 1 (0.2) | 1 (0.6) |
| Occupational status | |  |  |
|  | Missing – no. (%) | 21 (3.8) | 12 (7.6) |
| Psychological distress (SCL-5 score) | |  |  |
|  | Missing – no (%) | 20 (3.6) | 24 (15.3) |
| Type of stay | |  |  |
|  | Missing – no. (%) | 1 (0.2) | 0 (0.0) |
