# Supplementary figures and images for: Missed opportunities: the detection and management of at-risk drinking and illicit drug use in acutely hospitalized patients
Source: Adv Drug Alcohol Res. 2025 Mar 5;5:14149. doi: 10.3389/adar.2025.14149 (PMC11919628; doi:10.3389/adar.2025.14149)

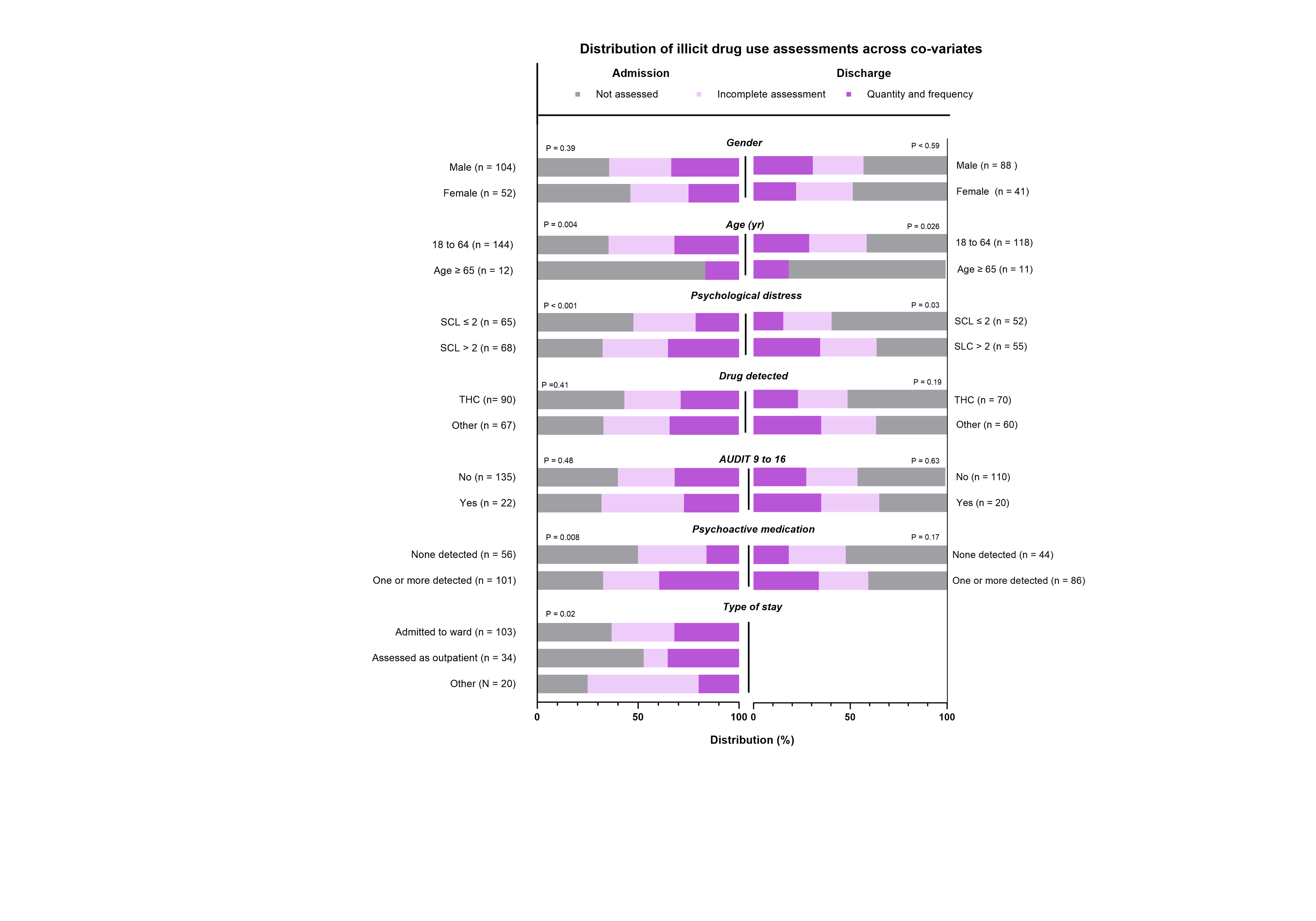

Supplement: Supplementary file 2 [file Image1.TIF]
